# Borneol, a novel agent that improves central nervous system drug delivery by enhancing blood–brain barrier permeability

**DOI:** 10.1080/10717544.2017.1346002

**Published:** 2017-07-07

**Authors:** Qun-Lin Zhang, Bingmei M. Fu, Zhang-Jin Zhang

**Affiliations:** a School of Pharmacy, Anhui Medical University, Hefei, China;; b Department of Biomedical Engineering, The City College of the City University of New York, NY, USA;; c School of Chinese Medicine, LKS Faculty of Medicine, The University of Hong Kong, Hong Kong, China

**Keywords:** Borneol, blood–brain barrier, permeability, drug delivery, CNS drugs

## Abstract

The clinical application of central nervous system (CNS) drugs is limited by their poor bioavailability due to the blood–brain barrier (BBB). Borneol is a naturally occurring compound in a class of ‘orifice-opening’ agents often used for resuscitative purposes in traditional Chinese medicine. A growing body of evidence confirms that the ‘orifice-opening’ effect of borneol is principally derived from opening the BBB. Borneol is therefore believed to be an effective adjuvant that can improve drug delivery to the brain. The purpose of this paper is to provide a comprehensive review of information accumulated over the past two decades on borneol’s chemical features, sources, toxic and kinetic profiles, enhancing effects on BBB permeability and their putative mechanisms, improvements in CNS drug delivery, and pharmaceutical forms. The BBB-opening effect of borneol is a reversible physiological process characterized by rapid and transient penetration of the BBB and highly specific brain regional distribution. Borneol also protects the structural integrity of the BBB against pathological damage. The enhancement of the BBB permeability is associated with the modulation of multiple ATP-binding cassette transporters, including P-glycoprotein; tight junction proteins; and predominant enhancement of vasodilatory neurotransmitters. Systemic co-administration with borneol improves drug delivery to the brain in a region-, dose- and time-dependent manner. Several pharmaceutical forms of borneol have been developed to improve the kinetic and toxic profiles of co-administered drugs and enhance their delivery to the brain. Borneol is a promising novel agent that deserves further development as a BBB permeation enhancer for CNS drug delivery.

## Introduction

Although considerable advancements have been made in drug delivery to the central nervous system (CNS), the clinical application of CNS drugs is still limited by their poor bioavailability due to the blood–brain barrier (BBB) (Pardridge, 2005; Denora et al., 2009). The special anatomic features of the BBB protect the CNS from toxins and variations in blood composition, and maintain the constancy of the brain’s micro-environment (Abbott & Friedman, 2012). The BBB is a physical and metabolic barrier that restricts the penetration of molecules (Pardridge, 2005). A key determinant of the kinetic and therapeutic properties of CNS drugs is their ability to cross the BBB (Fu, 2012). The temporary opening of the paracellular pathway of the BBB is believed to be an effective strategy to enhance drug delivery to the brain (Pardridge, 2005; Shi et al., 2014a,b).

**Figure 1.**
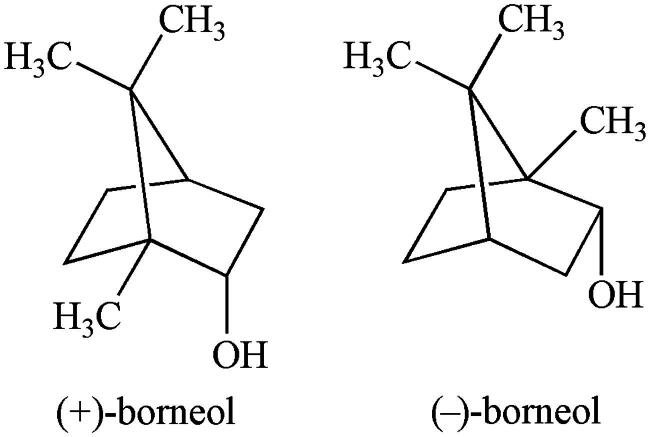
Molecular structures of borneol.

In traditional Chinese medicine (TCM), a group of drugs is specifically used for resuscitative purposes to restore consciousness in cases of coma, heart attack, stroke, traumatic brain injury and other brain-related emergency conditions. Most TCM resuscitation agents are derived from aromatic mineral and animal materials and are referred to as aromatic ‘orifice-opening’ agents (Wang et al., 2014). Borneol (*Bing-Pian* or *Long-Nao*) is a representative TCM resuscitation drug that has been used in clinical practice more than 1500 years. Borneol (C_10_H_18_O, molecular weight 154.25) is a highly lipid-soluble bicyclic monoterpene with fragrant odor and pungent and bitter tastes (Figure 1). Over the past two decades, this traditional agent has attracted increasing attention as a novel agent that can easily and rapidly cross the BBB due to its low molecular mass and high lipid solubility (Xiong et al., 2013; Wang et al., 2014). Several studies have been done to explore the ‘orifice-opening’ effects of borneol and their underlying mechanisms (Xiong et al., 2013; Wang et al., 2014). The objective of this paper is to provide a comprehensive review of borneol, including its chemical features, sources, toxic and kinetic profiles, enhancing effects on the BBB permeability and their putative mechanisms, improvement in CNS drug delivery and pharmaceutical forms.

## Sources

Borneol’s sources include natural extracts and artificial synthesis. One natural form of borneol is dextrorotatory borneol (*endo*-(1 *R*)-1,7,7-trimethyl-bicyclo[2.2.1] heptan-2-ol, (+)-borneol), which traditionally originated from the resin of *Dipterocarpus aromatica* Gaertn. f., a plant that grows in Southeast Asia. Due to its rarity, the essential oil extracted from the fresh branches and leaves of *Cinnamomum camphora* (L.) Presl. has been widely used as a substitute. Another natural form of borneol is levorotary borneol (*endo*-(1 *S*)-1,7,7-trimethyl-bicyclo [2.2.1] heptan-2-ol, (–)-borneol), which is extracted from fresh leaves of *Blumea balsamifera* (L.) DC., a traditional medicinal plant commonly used by the *Miao* minority in China (Wang et al., 2014). Synthetic borneol is an optically inactive (±)-borneol that is a mixture of (±)-borneol and isoborneol and is obtained via chemical transformation of camphor and turpentine oil. Camphor is the principal metabolite of borneol (Jiang et al., 2008). Natural and synthetic borneol and their metabolite camphor can be detected with gas chromatography and mass spectrometry (Cheng et al., 2013). For clinical use, the purity of (+)- and (–)-borneol must be no less than 96.0 and 85.0%, respectively, whereas the purity of (±)-borneol should be no less than 55.0% (State Pharmacopoeia Committee, 2015).

## Toxicological profile

An ideal enhancer of the BBB permeability should be stable, nontoxic, nonirritant and compatible with other compounds (Banks, 2009). Natural borneol has been extensively used in aromatherapy and in natural and cosmetic products because of its low toxicity compared to synthetic borneol, which toxicity is relatively high as it degrades slowly during storage, and noxious camphor levels may vary from 45% to as high as 97% (Zeng & He, 2004; Xiong et al., 2013; Wang et al., 2014). However, due to its price advantage, synthetic borneol is often used to replace natural borneol.

Median lethal dose (LD_50_) is the dose of a toxic agent that is sufficient to kill 50% of a population of animals. Oral LD_50_ of borneol are 300–5800 mg/kg in rodents and 3200 mg/kg in rabbits (https://www.ncbi.nlm.nih.gov/pccompound). The IC_50_ values, defined as the median inhibitory concentration that causes 50% cell death, are 2.5–3.0 mM for rat hepatocytes, 2750 μM for HepG2 cells, 2250 μM for Caco-2 cells and 1500 μM for VH10 cells (Slameňová et al., 2009). Cytotoxicity of borneol at concentrations above IC_50_ manifests as genotoxicity with DNA damage. However, repeated administration of borneol at daily concentrations of 17.14 mg/kg and 34.28 mg/kg for 7 days reduced H_2_O_2_-induced DNA damage in hepatocytes and testicular cells, respectively (Horváthová et al., 2009), suggesting a difference in toxic response between acute and chronic borneol regimens. No significant toxic effects of borneol were observed in mouse oral fibroblasts at concentrations of 18.75–150 μg/mL (Dai et al., 2009), corneal tissue at a concentration of 0.1% (Yang et al., 2009), or in rat thymocytes at a concentration of 5 μg/mL (Cherneva et al., 2012). The doses of natural and synthetic borneol recommended for clinical use are 0.3–0.9 g and 0.15–0.3 g, respectively (State Pharmacopoeia Committee, 2015).

## Kinetic characteristics of borneol in peripheral organs and the brain

Oral administration is most common in the clinical use of borneol. Borneol is absorbed rapidly into the brain and can be determined in the brain at the same concentration as in the blood within 5 min of oral administration, which suggests that it can rapidly cross the BBB and enter brain tissues (Liang et al., 1993). The maximum concentration (*C*_max_) in the brain is reached within 1 h after dosing (Liang et al., 1993; Li et al., 2012). Borneol has a relatively lower blood albumin binding constant [2.4 × 10^3^ (mol/L)^−1^] and rate (59.5%) than most other orally administered CNS drugs, for which brain bioavailability is generally low due to the hepatic first-pass effect, high plasma protein binding and hindrance by the BBB (Hu & Chen, 2009). In mice, a single oral dose of borneol accumulates in organs in the order of liver > brain > kidney > heart > spleen > muscle > lung, which confirms its considerably higher bioavailability in the brain than in most other organs examined (Huang et al., 2009). The distribution of borneol in the brain shows regional specificity, with the concentration highest in the cortex, moderate in the hippocampus and hypothalamus and lowest in the striatum (Yu et al., 2013a).

Intranasal drug delivery can avoid gastrointestinal destruction and hepatic first-pass metabolism, resulting in rapid onset of effect and high brain bioavailability. It is therefore considered to be an effective approach for borneol administration. One study has compared the kinetic profile of borneol administered via oral, intranasal, and intravenous route (Zhao et al., 2012); the results are summarized in Table 1. The blood absolute bioavailability associated with the intranasal route is approximately twice that of the oral route and is comparable to that of the intravenous route in mice (Zhao et al., 2012). Similar results were also obtained in rats (Song et al., 2012). However, the brain *C*_max_ and brain/blood drug ratio were similar for the intranasal and oral routes (Zhao et al., 2012), which suggests that the delivery rate of borneol into the brain does not differ between the oral and intranasal routes.

**Table 1.** Comparisons of kinetic profile of orally, intranasally and intravenously administered borneol in mice using the noncompartmental model.

	i.v.	i.n.	p.o.
Plasma^a^			
* C*_max_ (μg/mL)	68.0 ± 8.4	25.9 ± 5.0**	15.6 ± 2.3**
* T*_max_ (min)	1.0	3.0	10.0
* *AUC_0–120 min_ (μg·mL^−1^·min)	632.3 ± 50.8	573.4 ± 77.7	271.8 ± 37.4**
* *MRT_0–120 min_ (min)	12.2 ± 1.6	28.2 ± 1.4**	17.9 ± 2.1**
* F* (%)	100	90.7	43.0
Brain			
* C*_max_ (μg/g)	43.0 ± 5.1	9.0 ± 1.4**	8.5 ± 2.0**
* T*_max_ (min)	1.0	3.4	10.0
* *AUC_0–120 min_ (μg·g^−1^·min)	505.3 ± 82.9	345.5 ± 70.0*	194.0 ± 23.5**
* *MRT_0–120 min_ (min)	20.5 ± 8.8	49.0 ± 8.3**	43.6 ± 17.1**
* Re* (%)	100	68.4	38.4
* Te* (%)	79.9	60.3	71.4
* *DTI	1.0	0.8	0.9

This table is modified based on Zhao et al. (2012). Data are expressed as mean ± SD (*n* = 5). *p < 0.05, **p < 0.01: versus intravenous group, using one-way analysis of variance (ANOVA). i.v.: intravenous administration; i.n.: intranasal administration; p.o.: oral administration; *C*_max_: calculated maximum concentration; *T*_max_: time corresponding to *C*_max_; AUC_0–120 min_: partial area under the curve from 0 to 120 min; MRT_0–120 min_: mean residence time from 0 to 120 min; *F*: absolute bioavailability; *Re*: relative brain targeted coefficient; *Te*: brain/blood drug ratio; DTI: drug target index.

## Improvement of drug delivery to the brain

### Evidence for improved drug delivery to the brain

A large body of evidence has confirmed the effects of borneol in improving drug delivery to the brain (Table 2). Systemic co-administration with borneol produced a 26–197% increase in brain *C*_max_ and AUC values of the co-administered agents tetramethylpyrazine (ligustrazine) (Liu et al., 2008; Xiao et al., 2008), nimodipine (Wu et al., 2014), gastrodin (Cai et al., 2008), rifampicin (Wu et al., 2004), geniposide (Dong et al., 2012) and hyperforin (Yu et al., 2011b) compared to the agents alone, indicating that the addition of borneol enhances the transportation and bioavailability of drugs in the brain. Furthermore, the brain bioavailability of geniposide was approximately five times greater when it was given by intranasal co-administration with borneol than when the drugs were given by intragastric co-administration (Lu et al., 2012).

**Table 2.** Borneol improves kinetic profile of co-administered agents in the brain.

			*C*_max_ (μg/g)		AUC (μg/mL/min)		
Borneol (mg/kg)	Co-administered agents (mg/kg)	Sample	alone	combined	alone	combined	Ref.
30, i.g.	TMPP, 37.5, i.g.	Mouse brain homogenate	2.83	4.38	103.54	168.15	Xiao et al., (2008)
0.9, i.n.	TMPP, 5, i.n.	Rat brain homogenate	10.92	14.16	1.95^a^	2.80^a^	Liu et al., (2008)
250, i.g.	Nimodipine, 2, i.v.	Mouse brain homogenate			21.86	28.42	Wu et al., (2014)
400, i.g.	Gastrodin, 200, i.g.	Mouse brain homogenate	0.50	0.63	50.12	104.67	Cai et al., (2008)
200, i.g.	Geniposide, 300, i.v.	Rat brain homogenate	19	37	810	2410	Dong et al., (2012)
600, i.g.	Rifampicin, 182, i.g.	Mouse brain homogenate	2.0	5.1	285.1	569.5	Wu et al., (2004)
300/600, i.g.	Hyperforin, 200, i.g.	Rat brain homogenate	0.13^b^	0.23–0.24	13.8	19.5–19.7	Yu et al., (2011b)
300, i.g.	Levodopa, 416, i.g.	Rat CSF	4.29^b^	9.00^b^	2587.5	2890.4	Xian et al., (2013)
300, i.g.	Puerarin, 62.5, i.v.	Rat cortex			117.6	341.8	Gao et al., (2010)
300, i.g.	Edaravone, 3.75, i.v.	Rat cortex			50.83	70.29	Gao et al., (2010)
30, i.v.	Kaempferol, 25, i.v.	Rat hippocampus	0.11	0.35	13.06	28.57	Zhang et al., (2015)
186, i.g.	Meropenem, 208, i.p.	Rat striatum	1.20	2.25	122.56	233.25	Xin et al., (2014)

AUC: area under the curve; *C*_max_: maximum concentration; CSF: cerebrospinal fluid; i.g.: intragastrical; i.n.: intranasal; i.p.: intraperitoneal; i.v.: intravenous; TMPP: tetramethylpyrazine phosphate (ligustrazine).

^a^ μg/mL/h.

^b^ μg/mL.

Borneol co-administered orally with levodopa, a metabolic precursor that is converted to dopamine in the brain and mainly used for the treatment of Parkinson’s disease, increased its *C*_max_ by 115% and AUC by 12% in rat cerebrospinal fluid (CSF) (Xian et al., 2013). This finding raises the possibility that, like carbidopa, which is always given in combination with levodopa to prevent the metabolism of levodopa in the periphery, borneol could serve as an enhancer to facilitate the brain distribution and bioavailability of levodopa, thus reducing the required dosage and related side effects.

### Brain-regional specificity of the improvement in CNS drug delivery

Microdialysis studies have revealed that when geniposide was given in combination with borneol, the accumulation and bioavailability of geniposide largely improved in the hippocampus and the hypothalamus, but was suppressed in the cortex and unaltered in the striatum compared to geniposide alone (Yu et al., 2013a). Similar region-specific enhancement by borneol was also observed for puerarin and edaravone in the cortex (Gao et al., 2010), kaempferol in the hippocampus (Zhang et al., 2015), meropenem in the striatum (Xin et al., 2014) and ligustrazine in the hypothalamus and the striatum (Yu et al., 2016). This may be directly associated with the brain region-specific distribution of borneol, suggesting that borneol’s enhancement of CNS drug delivery varies in different brain regions. The brain region-targeted effect of borneol could help co-administered agents to gain greater access to specific brain regions.

### Dose- and time-dependent improvement in CNS drug delivery

Systemic co-administration with borneol in low doses ranging from 0.05 to 2.0 g/kg facilitated the delivery of geniposide to the rat brain, but high doses of 2.0–4.0 g/kg suppressed geniposide delivery compared to administration without borneol (Dong et al., 2012). Borneol also increased the quantity and velocity of geniposide permeating the brain and the effect reached the most obvious at 15 min after both agents were given simultaneously via the intragastric route in rats (Yu et al., 2012). Similar results were observed for borneol co-treatment with gastrodin in mice (Cai et al., 2008). The enhancing effects on the brain bioavailability of gastrodigenin, an active metabolite of gastrodin, reached a peak level with borneol doses of 200–600 mg/kg, but were attenuated with doses above 600 mg/kg (Cai et al., 2008). When borneol was given at different intervals, a robust enhancing effect on brain gastrodigenin bioavailability was achieved within 40 min (Cai et al., 2008). Likewise, oral co-treatment with borneol at doses of 15–90 mg/kg, but not higher doses, produced a proportionally greater increase in plasma and brain concentration of tetramethylpyrazine phosphate (TMPP) in mice compared to treatment without borneol, particularly in the early period (Xiao et al., 2007).

These studies indicate that borneol enhances CNS drug delivery in a dose- and time-dependent manner. The optimal dosing regimen of borneol that rapidly produces the maximum enhancement of brain delivery without unfavorable side effects varies from one co-administered drug to another and should be determined individually.

## Borneol’s regulation of BBB permeability and putative mechanisms

### *In vivo* and *in vitro* evidence

An early dynamic computed tomographic study found that arterial injection of borneol significantly enhanced the transport of diatrizoate, a water-soluble contrast agent used during radiography, into the rabbit brain (Wang et al., 1992). Since then, a growing body of evidence has confirmed the enhancing effects of borneol on BBB permeability (Zhai & Shen, 2010). Evans blue is the marker most commonly used to investigate BBB integrity and permeability because it binds to albumin, a high-molecular weight protein that cannot cross the BBB if the barrier is structurally and functionally intact (Saunders et al., 2015). Experimental animal studies have clearly demonstrated that systemic borneol significantly increased the amount of Evans blue entering the brain (Yu et al., 2011a; Zhai & Shen, 2010). Furthermore, repeated systemic borneol significantly widened tight junctions and increased the number of void structures between the endothelial cells of the BBB examined with transmission electron microscopy in the rat brain (Yu et al., 2013b).

The permeability-enhancing effect of borneol has been further confirmed in *in vitro* experiments. Madin–Darby canine kidney epithelial (MDCK) cell culture is a commonly used cell model of the BBB because it displays several structural similarities to the BBB, including intercellular tight junctions and other related subcellular components (Rodriguez-Boulan et al., 2005). Borneol-containing serum added to MDCK cells resulted in the loosening of intercellular tight junction and in an increase in number and enlargement of pinocytosis vesicles (Chen & Wang, 2004). The loosening of tight junctions and enhanced pinocytosis, respectively, was observed at 4 and 24 h after treatment with borneol but disappeared at 24 h after removal of the borneol-containing serum (Chen & Wang, 2004), which indicates that borneol enhancement of BBB permeability is reversible and transient.

Inducible nitric oxide synthase (iNOS) is expressed only after cell activation in response to pathological insults and often serves as a biomarker to differentiate between physiological and pathophysiological actions of nitric oxide (NO) (Kröncke et al., 1998). The level of iNOS expression in brain microvascular endothelial cells was strikingly increased in rats with brain traumatic injury but was unchanged in intact rats (Zhao et al., 2002). Oral borneol given 6 h after head injury markedly attenuated the increased iNOS expression in injured rats but did not affect the level of expression in intact rats (Zhao et al., 2002). It is clear that borneol-induced opening of the BBB is a reversible physiological process rather than a consequence of pathological damages. Indeed, borneol protects the structural integrity of the BBB by modulating vascular endothelial growth factor in rats with cerebral ischemia-reperfusion injury (Ni et al., 2011). Borneol combined with ferulic acid, an agent often used to treat vascular diseases and to prevent thrombosis, suppressed the abnormally increased BBB permeability caused by cerebral ischemia in mice (Chen et al., 2010). Borneol also improved cell membrane fluidity in MDCK cells (Chen et al., 2014a) and in human nasal epithelial cells (Chen et al., 2014b).

Taken together, these findings show that borneol exerts a biphasic regulatory effect on BBB permeability, i.e. reversibly opening the BBB under physiological conditions, but protecting the structural integrity of the BBB against pathological damage.

### Possible mechanisms involved in the permeability-enhancing effects

**Figure 2.**
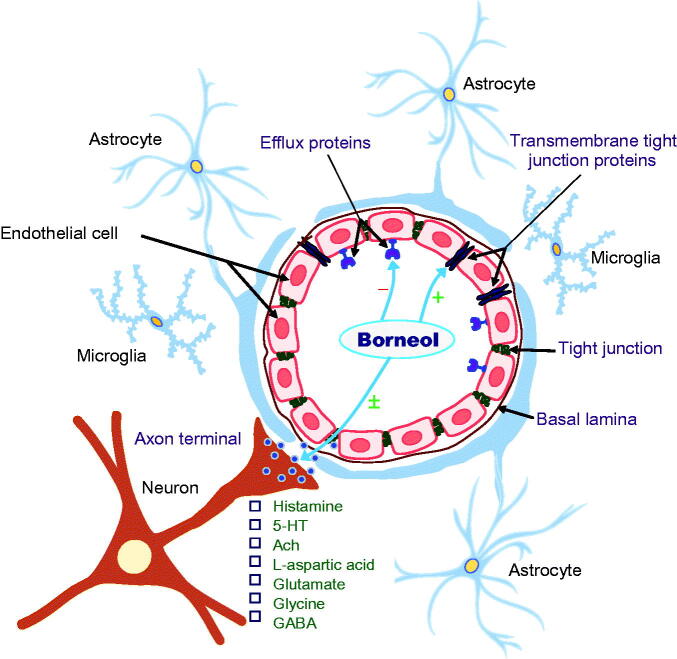
The permeability-enhancing effects of borneol may be achieved mainly via three mechanisms: the inhibition of efflux protein function; the enhancement of transmembrane tight junction protein; and predominant enhancement of vasodilatory neurotransmitters. (−), inhibitory effects; (+), enhancing effects; (±) biphasic regulatory effects.

The permeability-enhancing effects of borneol are closely associated with the inhibition of efflux protein function, the enhancement of transmembrane tight junction protein and predominant enhancement of vasodilatory neurotransmitters (Figure 2).

ATP-binding cassette (ABC) transporters are a novel family of ATP-dependent drug efflux proteins of the cell membrane that pump various foreign substances out of cells. P-glycoprotein (P-gp), also known as multidrug resistance protein, is a key member of the ABC transporter family that plays a crucial role in protecting BBB integrity (Ambudkar et al., 1999). Other ABC transporters are also extensively involved in the regulation of BBB function (Löscher & Potschka, 2005). Both *in vitro* and *in vivo* studies have shown that borneol inhibited the expression of P-gp and other ABC transporters, including multidrug resistance protein 1 (Mrp1), 1a (Mdr1a) and 1 b (Mdr1b), resulting in an increase in the amount of Evans blue and ABC transporter markers rhodamine 123 (Rh123), verapamil and digoxin that enter the brain (Fan et al., 2015; Yu et al., 2011a; Zhang et al., 2011; Yu et al., 2013a,b). These ABC transporter markers have low brain permeability under physiological conditions because they are recognized and inhibited by efflux transporters at the BBB (Perloff et al., 2003). Similar inhibitory effects of borneol on P-gp were also observed in MDR1-MDCK cells originated by transfecting MDCK cells with the MDR1 gene, which encodes for P-gp (Chen et al., 2013). It appears that the permeability-enhancing effect of borneol is at least in part derived from its suppression of P-gp and other related efflux protein functions.

Tight junction proteins, particularly transmembrane proteins such as claudins and occludin, play a principal role in defining epithelial cell polarity, regulating paracellular permeability and conferring barrier function (Paris et al., 2008). The translocation of claudin-5 and occludin from the cell membrane to the cytoplasm was present 30 min after the initiation of borneol treatment and reached peak levels at 1 h, but returned to the normal pattern 8 h after treatment in endothelial cells in the blood-optic nerve barrier of rats (Jin et al., 2011). It is likely that borneol exerts its permeability-enhancing effects by reversibly disassembling tight junction proteins in the BBB.

BBB permeability is directly and indirectly regulated by multiple neurotransmitters, particularly histamine (Sarker et al., 1998), serotonin (Cohen et al., 1996), N-methyl-d-aspartate (NMDA) (Meng et al., 1995; Neuhaus et al., 2011) and acetycholine (Abbruscato et al., 2002). Activation of these transmitters produces cerebral vasodilation via nitric oxide and receptors on perivascular astrocytes and microvessel endothelial cells of the BBB.

Although there is no direct evidence to confirm that the enhancing effects of borneol on BBB permeability are related to its modulation of vasodilatory neurotransmitters, systemic borneol was found to increase the levels of histamine and serotonin in the hypothalamus (Li et al., 2004, 2006) and levels of l-aspartic acid, glutamate, glycine and γ-aminobutyric acid (GABA) in the corpus striatum of rats (Zhang et al., 2012). Moreover, the magnitude of the increase in excitatory amino acid levels was considerably greater than that of the increase in inhibitory amino acids in the whole brain, resulting in a transient elevation in the excitation ratio (excitatory amino acids versus inhibitory amino acids) (Li et al., 2012). Natural and synthetic borneol enhanced the actions of GABA via GABA_A_ receptors in *Xenopus laevis* oocytes (Granger et al., 2005) and also modulated nicotinic acetylcholine receptor-mediated effects in a noncompetitive manner, without affecting the intracellular calcium level in bovine adrenal chromaffin cells (Park et al., 2003).

There is reason to postulate that the transient and reversible effects of borneol in enhancing BBB permeability may be related to its temporary and predominant enhancement of vasodilatory neurotransmitters.

## Pharmaceutical forms of borneol

To improve the kinetic and toxic profiles of co-administered drugs and enhance delivery to the brain, various pharmaceutical forms of borneol have been developed.

One recent study reported a novel brain glioma-targeting delivery system called FA-BO-PAMAM/DOX, which was prepared from borneol (BO)-modified PAMAM G5 dendrimer in conjugation with doxorubicin (DOX) and folic acid (FA) (Xu et al., 2016). Modification with borneol efficiently boosted BBB permeability, prolonged half-life time, increased the focal accumulation and bioavailability of DOX and augmented anti-tumor efficacy compared to the formulation without borneol. Furthermore, modification with borneol largely reduced cytotoxicity.

Another brain tumor-targeting delivery system modified with borneol is the lipid-protein nanocomplex BP-liprosome, in which borneol (B) and paclitaxel (P) are co-encapsulated by liprosomes (Tang et al., 2015). The nanocomplex is only approximately 108 nm, and it has high entrapment efficiencies of 86% for borneol and 90% for paclitaxel. BP-liprosomes has a longer release profile and higher accumulation in focal brain tumor lesions than that without borneol modification. It exhibits robust anti-tumor efficacy, with 86% versus 62% of liprosomes conjugated only paclitaxel, and 49% of paclitaxel solutions (Tang et al., 2015).

Borneol can also be incorporated in ganciclovir-loaded solid lipid nanoparticles (SLNs) using a modified microemulsion method (Ren et al., 2013). Borneol-modified SLNs significantly increased the brain distribution of ganciclovir compared to a ganciclovir injection and SLNs without borneol modification, indicating that borneol-modified SLNs are an efficient delivery system for transporting drugs to the brain (Ren et al., 2013).

Huperzine A is a naturally occurring compound that may have benefits in the treatment of Alzheimer’s disease and other cognitive impairments. Co-incubation with borneol increased the uptake of Huperzine A loaded aprotinin-modified nanoparticles by capillary endothelial cells (Zhang et al., 2013). Systemic co-administration of borneol further augmented the brain-targeting efficiency and cognition-improving effects of aprotinin-modified nanoparticles in rats (Zhang et al., 2013).

High doses of borneol often cause stomach irritation. The co-loading of borneol and gastrodin, a principal bioactive ingredient of the Chinese herbal medicine Rhizoma Gastrodiae (Tian-Ma), into sustained-release solid dispersions can largely reduce gastric mucosal irritation caused by borneol without sacrificing its targeting efficiency (Cai et al., 2014). Borneol and gastrodin co-loaded sustained-release solid dispersions were prepared using ethylcellulose as a sustained-release matrix and hydroxy-propyl methylcellulose as a retarder. Sustained-release technology appears to be an effective approach to minimizing stomach irritation while preserving sufficient transport capacity for brain-targeted delivery of orally administered borneol (Cai et al., 2014).

## Conclusions

Borneol is a naturally occurring compound in a class of ‘orifice-opening’ agents used in TCM for resuscitative purpose. Several lines of evidence confirm that the ‘orifice-opening’ effects of borneol are mainly derived from BBB opening. The BBB-opening effect of borneol is a reversible physiological process characterized by rapid and transient penetration through the BBB and highly specific brain regional distribution. Borneol also protects the structural integrity of the BBB against pathological damages. The enhancing effects of borneol on BBB permeability are associated with the modulation of ABC transporters, including P-gp, tight junction proteins, and the predominant enhancement of vasodilatory neurotransmitters. Systemic co-administration with borneol improves drug delivery to the brain in a region-, dose- and time-dependent manner. Various pharmaceutical forms of borneol, such as FA-BO-PAMAM/DOX, BP-liprosome and solid lipid nanoparticles, have been developed to improve the kinetic and toxic profiles of co-administered drugs and enhance their delivery to the brain.
